# Large-scale analysis of antisense transcription in wheat using the Affymetrix GeneChip Wheat Genome Array

**DOI:** 10.1186/1471-2164-10-253

**Published:** 2009-05-29

**Authors:** Tristan E Coram, Matthew L Settles, Xianming Chen

**Affiliations:** 1 US Department of Agriculture – Agricultural Research Service, and Washington State University, Department of Plant Pathology, Pullman, WA 99164-6430, USA; 2 Department of Molecular Biosciences, Washington State University, Pullman, WA, 99164-4234, USA; 3 US Department of Agriculture – Agricultural Research Service, and North Carolina State University, Department of Crop Science, Campus Box 7620, Raleigh, NC, 27695-7620, USA

## Abstract

**Background:**

Natural antisense transcripts (NATs) are transcripts of the opposite DNA strand to the sense-strand either at the same locus (*cis*-encoded) or a different locus (*trans*-encoded). They can affect gene expression at multiple stages including transcription, RNA processing and transport, and translation. NATs give rise to sense-antisense transcript pairs and the number of these identified has escalated greatly with the availability of DNA sequencing resources and public databases. Traditionally, NATs were identified by the alignment of full-length cDNAs or expressed sequence tags to genome sequences, but an alternative method for large-scale detection of sense-antisense transcript pairs involves the use of microarrays. In this study we developed a novel protocol to assay sense- and antisense-strand transcription on the 55 K Affymetrix GeneChip Wheat Genome Array, which is a 3' *in vitro *transcription (3'IVT) expression array. We selected five different tissue types for assay to enable maximum discovery, and used the 'Chinese Spring' wheat genotype because most of the wheat GeneChip probe sequences were based on its genomic sequence. This study is the first report of using a 3'IVT expression array to discover the expression of natural sense-antisense transcript pairs, and may be considered as proof-of-concept.

**Results:**

By using alternative target preparation schemes, both the sense- and antisense-strand derived transcripts were labeled and hybridized to the Wheat GeneChip. Quality assurance verified that successful hybridization did occur in the antisense-strand assay. A stringent threshold for positive hybridization was applied, which resulted in the identification of 110 sense-antisense transcript pairs, as well as 80 potentially antisense-specific transcripts. Strand-specific RT-PCR validated the microarray observations, and showed that antisense transcription is likely to be tissue specific. For the annotated sense-antisense transcript pairs, analysis of the gene ontology terms showed a significant over-representation of transcripts involved in energy production. These included several representations of ATP synthase, photosystem proteins and RUBISCO, which indicated that photosynthesis is likely to be regulated by antisense transcripts.

**Conclusion:**

This study demonstrated the novel use of an adapted labeling protocol and a 3'IVT GeneChip array for large-scale identification of antisense transcription in wheat. The results show that antisense transcription is relatively abundant in wheat, and may affect the expression of valuable agronomic phenotypes. Future work should select potentially interesting transcript pairs for further functional characterization to determine biological activity.

## Background

Natural antisense transcripts (NATs) are defined as transcripts of the opposite DNA strand to the sense-strand either at the same locus (*cis*-encoded) or a different locus (*trans*-encoded). The first NATs were detected in viruses, followed by prokaryotes and then eukaryotes. For an excellent review of current NAT knowledge, please refer to Lapidot and Pilpel [1]. NATs usually possess a negative regulatory effect and can affect gene expression at multiple stages including transcription, RNA processing and transport, and translation [2,3]. Thus, NATs may be involved in the regulation of varying biological functions such as the adaptation to stresses and development. NATs are involved in RNA interference [4,5], methylation [6] and genomic imprinting [7]. NATs give rise to sense-antisense transcript pairs that were once considered as rare, but the number identified has escalated greatly with the availability of DNA sequencing resources and public databases. For example, 22% of annotated genes in the fruit fly genome are reported to overlap as transcript pairs [8], and more than 20% of human transcripts may form sense-antisense transcript pairs [9]. In plants, few sense-antisense transcript pairs had been reported until recent large-scale studies in rice [10,11] and *A. thaliana *[12,13]. In the rice study, full-length cDNA data revealed that approximately 7% of transcripts formed sense-antisense transcript pairs [10]. In these plant studies, the alignment of full-length cDNAs and expressed sequence tags (ESTs) to the genome sequence was used to identify the sense-antisense transcript pairs, which is limited to the detection of *cis*-encoded pairs. In wheat, antisense transcripts have been discovered from serial analysis of gene expression (SAGE) tags of developing grain [14], where it was reported that 25.7% of forward (sense) tags had a matching reverse (antisense) tag, which indicated widespread antisense transcription in wheat.

An alternative method for large-scale discovery of sense-antisense transcript pairs involves the use of microarrays. In the first study of this type, Yelin *et al*. [15] used a strand-specific oligonucleotide probe array to detect antisense transcription in human cell lines. A study in mouse using a custom oligonucleotide array to assay the expression of 1,947 known sense-antisense transcript pairs has also been reported [16]. However, these studies required prior knowledge of the sense-antisense transcript pairs to enable the design of strand specific probes. To overcome this, Werner *et al*. [17] took advantage of the approximately 25% of incorrectly orientated probes on the Affymetrix GeneChip U74A and U74B 3'*in vitro *transcription (3'IVT) mouse arrays to detect novel antisense transcription in mouse brain and kidney tissues. The results showed that the commercial expression arrays were sensitive enough to detect antisense transcription, but because it cannot be assumed that current commercial arrays contain incorrectly orientated probes, this type of study could not be repeated. Subsequently, Ge *et al*. [18] developed a method called 'Antisense Transcriptome analysis using Exon array (ATE)' that used an altered target synthesis and labeling method that allowed both sense- and antisense-strand transcription to be assayed on Affymetrix Whole-Transcript Expression arrays (ie. Exon and Gene arrays). This protocol was successful but cannot be applied to the numerous Affymetrix (3'IVT) expression arrays, because these arrays are constructed with probes of the opposite strand to the Whole-Transcript Expression arrays, thus they use a different target labeling procedure altogether.

In the current study, we sought to develop a protocol that could be used to assay sense- and antisense-strand transcription on the Affymetrix GeneChip Wheat Genome array, which is a 3'IVT expression array. The 3'IVT expression arrays rely on *in vitro *transcription of double-stranded cDNA to both amplify and label the target cRNA before hybridization. The wheat array currently provides the most comprehensive coverage of the wheat genome for a microarray and is a commonly used resource for transcript expression studies [19,20] and hybridization-based DNA marker discovery [21]. This study is the first report of using a 3'IVT expression array to discover the expression of natural sense-antisense transcript pairs without relying on the presence of incorrectly oriented probes, and may be considered as proof-of-concept. By using alternative target preparation schemes, both the sense- and antisense-strand derived transcripts were labeled and hybridized to the Wheat Genome Array. To enable maximum discovery we selected five different tissue types for assay and used 'Chinese Spring' wheat genotype, since most of the GeneChip probe sequences were based on its genomic sequence. The functional annotation of detected wheat sense-antisense transcript pairs is discussed, as well as the performance and validation of the technique.

## Results

### Target preparation

**Figure 1:**
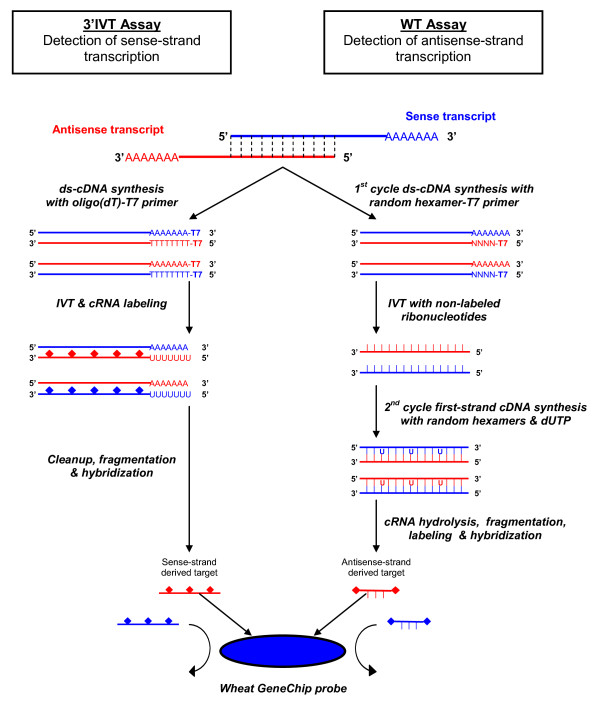
**Principles of the two target preparation methods used to assay both sense- and antisense-strand transcription**. A 5' (head-to-head) overlapping sense-antisense transcript pair is used as an example. The standard Affymetrix 3' *in vitro *transcription (3'IVT) assay was used to detect sense-strand transcription, while a modified Affymetrix Whole Transcript (WT) assay was used to detect antisense-strand transcription.

Total RNA was extracted from five 'Chinese Spring' tissue types (germinated seed, shoot, flag leaf, spike pre-anthesis and spike post-anthesis; see materials and methods). In addition to maximizing discovery, these tissue types were also selected to align with the predominant tissues used for wheat EST sequencing efforts, including the International Wheat Genome Sequencing Consortium (IWGSC). All samples were of excellent quality as assessed by gel electrophoresis and spectrophotometry. The total RNA samples were mixed at equal concentrations before target preparation. The assay of sense-strand transcription followed the regular scheme as for all Affymetrix 3'IVT GeneChips (see materials and methods). However, to assay antisense-strand transcription, the Affymetrix Whole Transcript (WT) Sense Target Labeling Assay was used, which was designed specifically for use on Whole-Transcript Expression arrays. The WT target preparation method resulted in labeling the opposite strand to the 3'IVT assay and was therefore used in this study to assess antisense-strand transcription (Figure 1). The mixed total RNA sample was used as starting material for both the 3'IVT and WT target preparation, and each hybridization was carried out once according to standard Affymetrix protocol for the Wheat Genome Array.

### Data analysis

**Figure 2:**
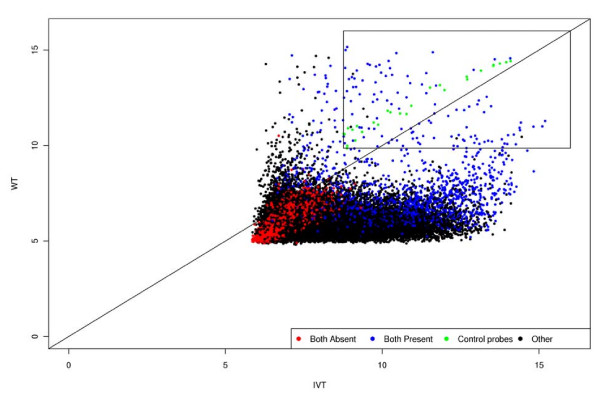
**Comparison of the 3'IVT (sense signal) and WT (antisense signal) array distributions**. The summarized log_2 _expression value for all probe sets (n = 61,127). Red spots indicate probe sets with absent MAS 5.0 PMA calls (*P *< 0.01) in both assays, black spots indicate probe sets called as present in one assay only, blue spots indicate probe sets with present calls in both assays, and green spots indicate the replicated spiked-in hybridization controls (*bioB, bioC, bioD *and *creX*). The black box shows the probe sets with expression values greater than the spiked-in hybridization controls in both assays, which were considered as positively hybridizing for this study.

Following hybridization and scanning, CEL files were analyzed to identify probe sets that showed successful hybridization in each of the 3'IVT and WT assay. The quality control metrics from the *affyQCreport *package [22] of Bioconductor [23] showed that data was of high-quality for both assays, but as expected the WT assay resulted in a lower percentage of detected transcripts (16.27%) than the 3'IVT assay (47.95%) using Affymetrix PMA (Present/Marginal/Absent) calls. Subsequently the relationship of array distributions (Figure 2) showed a skewing towards the 3'IVT assay, but it is clear that successful hybridization did occur in the WT assay. Figure 2 also showed that the spiked-in hybridization controls from Affymetrix (*bioB*, *bioC*, *bioD *and *creX*) produced similar signals from both assays although signals were slightly higher in the WT assay. The MAS 5.0 PMA calls and Robust Multi-array Average (RMA) summarized expression values were used to determine successful hybridization for each probe set. Because of differences in the two labeling methods, including starting amount of RNA and RNA amplification, the expression values of each array could not be validly compared. However, the PMA calls in combination with the expression values were used to determine positive hybridization to a particular probe set in each assay. This provided a qualitative measure of expression rather than quantitative, but for the purposes of this study which was to detect natural sense-antisense transcript pairs, this measure was satisfactory.

### Identifying sense-antisense transcript pairs

To determine a confident positive threshold for expression value in both assays, the expression values of spiked-in hybridization controls (*bioB*, *bioC*, *bioD *and *creX*) were used. Because these controls are spiked-in immediately before hybridization, they were expected to behave in the same way in both assays. The *bioB *control is spiked-in at the detection limit, while the others are spiked-in at staggered concentrations after *bioB*. Thus, the log_2 _expression value of *bioB *was considered the threshold for positive hybridization in each assay (Figure 2). Because the *bioB *probe set is replicated three times on the wheat GeneChip, the log_2 _expression value of the lowest individual probe set was used. For the 3'IVT assay this value was 8.76, and for the WT assay it was 9.86. These cut-off values were used in combination with the MAS 5.0 PMA calls and corresponding probability (*p*) values to detect successful hybridization in each assay. In both assays, a probe set must have firstly been called 'Present' with Wilcoxon rank sum test *p-*value < 0.01, and the RMA summarized log_2 _expression value must have been greater than 8.76 in the 3'IVT assay, and greater than 9.86 in the WT assay. This threshold cutoff identified 110 probe sets as positively hybridizing in both the 3'IVT and WT assays. In addition to the 110 probe sets, 8940 probe sets uniquely hybridized in the 3'IVT assay and 80 uniquely hybridized to the WT assay (potentially antisense-specific transcripts). Because the aim of this study was detect transcript pairs transcribed from both strands, we mainly focused on probe sets detected in both assays. These stringent detection criteria ensured that the probe sets left were highly expressed in both assays and could more reliably be considered as sense-antisense transcript pairs. In fact, the 80 antisense-specific probe sets could not necessarily be classified as antisense transcripts, because these may represent incorrectly orientated probes. Also, because the probes for a given transcript do not cover the entire sequence, there is a possibility for bias during hybridization. However, to form the basis of future studies these 80 probe sets were also given some attention.

### Annotation of probe sets

Each of the 110 candidate sense-antisense transcript pair probe sets were functionally annotated using HarvEST (Affymetrix Wheat1 Chip version 1.52). Gene Ontology (GO) was based on the TIGR rice genome annotation such that if a unigene possessed a significant (<1e-10) BLASTx match to rice, as identified in HarvEST, the corresponding GO terms for the rice protein were used, if available. Of the 110 probe sets 76 could be annotated (see Additional file 1), of which 46 (59%) were classified as involved in energy production ('Energy'), including several representations of ATP synthase, photosystem proteins and RUBISCO. To determine the significance of overrepresentation of the number of energy-related transcripts identified, a hypergeometric test of selected energy-related terms in the HarvEST annotated transcript description were used (see 'Methods'). For the transcripts identified as present in one or both hybridizations, energy-related terms were identified in 1831 of the 24578 transcripts (7.4%) that possessed a transcript description. Using the same search terms, 46 of the 76 annotated probe sets identified in this study possessed energy-related terms. Subsequently, energy-related transcripts were found to be significantly over-represented in this study with a *p*-value of 4.88 × 10^-37^. The diversity of the annotated probe sets is summarized in Table 1.

**Table 1:** Summary of the 76 successfully detected and annotated sense-antisense transcript pairs in both the 3'IVT and WT assay.

**Number of transcripts**	**Functional category**^**1**^	**Putative function**^**2**^
20	Metabolism: Protein biosynthesis	Ribosomal protein
10	Energy	ATP synthase
9	Energy	Photosystem I protein
5	Energy	Photosystem II protein
4	Energy	NAD(P)H-quinone oxidoreductase
4	Energy	RUBISCO large subunit
2	Cell death	Putative senescence-associated protein
2	Energy	Carbonic anhydrase
2	Energy	Cytochrome b subunit
2	Energy	Cytochrome c subunit
2	Energy	Photosystem Q(B) protein
2	Energy	RUBISCO small subunit
2	Transcription	Maturase K
1	Energy	Chlorophyll a/b binding protein
1	Energy	RUBISCO activase
1	Energy	NADH dehydrogenase
1	Homeostasis	Metallothionein
1	Metabolism: Secondary	Nicotianamine synthase
1	Metabolism: Secondary	Thioredoxin
1	Transcription	DNA-directed RNA polymerase
1	Transcription	Translation initiation factor
1	Transcription	Zinc finger protein
1	Transport	Yip1 domain containing protein

^1^Functional categories were based on the Munich Information Center for Protein Sequences classifications

^2^Putative function shows the best significant BLASTX database hit from HarvEST.

The 80 potential antisense-specific probe sets were also annotated as described for the transcript pairs. Of the 80 probe sets only 31 could be annotated (see Additional file 2), of which 10 (32%) were classified as involved in energy production ('Energy') including several representations of RUBISCO. Nine (28%) were involved in transcription ('Transcription') and included several DNA-directed RNA polymerase transcripts. However, the majority of antisense-specific transcripts were of unknown function.

### Strand-specific transcription validation

**Figure 3:**
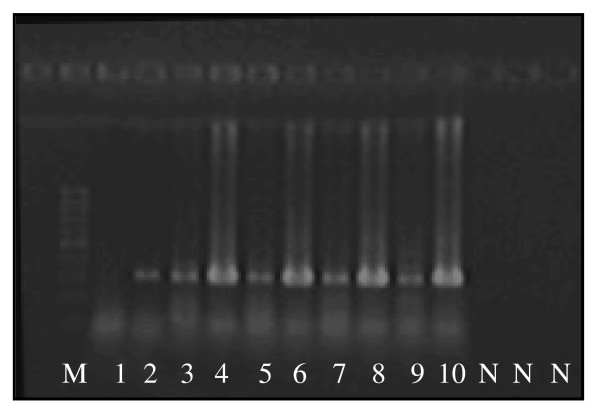
**Strand-specific RT-PCR for target Carbonic anhydrase (Ta.5227.1)**. Sense-strand transcription was detected only for germinated seed tissue, and both sense- and antisense-strand transcription for all other tissues. M: DNA marker, 1: Germinated seed antisense, 2: Germinated seed sense, 3: Shoot antisense, 4: Shoot sense, 5: Flag leaf antisense, 6: Flag leaf sense, 7: Spike pre-anthesis antisense, 8: Spike pre-anthesis sense, 9: Spike post-anthesis antisense, 10: Spike post-anthesis sense, N: Negative controls.

Ten probe sets selected to represent a range of functional categories were validated for sense- and antisense-strand transcription using strand-specific reverse transcription-PCR (RT-PCR). An example of the electrophoresis results is shown in Figure 3. Sense-strand transcription was detected for all 10 targets sets in each tissue except for the target RUBISCO activase in the 'Germinated seed' tissue (Table 2). In fact, the 'Germinated seed' tissue was most different to the other tissues and showed the least amount of antisense-strand transcription for the 10 targets. The 'Shoot', 'Flag leaf', 'Spike pre-anthesis' and 'Spike post-anthesis' tissues all showed the same pattern of sense- and antisense-strand transcription. These results indicate that antisense-strand transcription is likely specific to certain tissues and/or developmental stages, although not to a great extent in the 10 target transcripts analyzed in this study. Only one of the 10 targets (10%) did not show any antisense-strand transcription in any tissue, thus was not in agreement with the microarray results. However, this could be due to the position of the RT-PCR primer for amplifying the antisense-strand transcript. Because antisense-strand transcripts may not necessarily span the full-length of their complementary sense-strand transcript, the RT-PCR primer may have been targeted to a missing region in the antisense-strand transcript, thus the RT-PCR would fail.

**Table 2:** Validation of selected sense-antisense transcript pairs with strand-specific reverse transcription PCR (RT-PCR).

**Probe set ID**	**Putative function**^**1**^	**RT-PCR result**^**2**^									
		*Germinated seed*		*Shoot*		*Flag leaf*		*Spike pre-anthesis*		*Spike post-anthesis*	
		
		*S*	*A*	*S*	*A*	*S*	*A*	*S*	*A*	*S*	*A*

Ta.136.1	Thioredoxin	X		X	X	X	X	X	X	X	X
Ta.2752.2	RUBISCO small subunit	X		X	X	X	X	X	X	X	X
Ta.27660.1	RUBISCO activase			X		X		X		X	
Ta.5227.1	Carbonic anhydrase	X		X	X	X	X	X	X	X	X
TaAffx.128414.1	RUBISCO large subunit	X	X	X	X	X	X	X	X	X	X
TaAffx.128418.24	Putative senescence-associated protein	X	X	X	X	X	X	X	X	X	X
TaAffx.128757.1	Photosystem Q(B) protein	X	X	X	X	X	X	X	X	X	X
TaAffx.129824.1	Translation initiation factor	X	X	X	X	X	X	X	X	X	X
TaAffx.4530.1	ATP synthase alpha chain	X		X	X	X	X	X	X	X	X
TaAffx.4544.2	Cytochrome c biogenesis protein ccsA	X		X	X	X	X	X	X	X	X

^1^Functional categories were based on the Munich Information Center for Protein Sequences classifications and putative function shows the best significant BLASTX database hit from HarvEST.

^2^RT-PCR result shows detection of transcription (X) for the sense (S) strand and antisense (A) strand in each tissue.

## Discussion

This study reports on the first use of an Affymetrix GeneChip 3'IVT expression array for discovering both sense- and antisense-strand transcription. Through the adaptation of the Affymetrix WT assay, the antisense transcribed strand was successfully labeled and hybridized to the Wheat Genome Array, which allowed for the detection of natural sense-antisense transcript pairs. To our knowledge, the Wheat Genome Array does not contain any probes for known sense-antisense transcript pairs, thus the data from the hybridizations could not be standardized and/or normalized to a known sense-antisense transcript pair. Subsequently, a highly stringent data acceptance threshold was applied, based on PMA call and expression value cutoffs. This increased the confidence in detecting true antisense transcription. It is important to recognize the limitations of this study, which stem from the 'closed' nature of microarray systems. Because the Wheat Genome Array contains only known transcript sequences, the study is clearly limited to detection of transcript pairs that are present on the array. Further, the probes for each transcript are biased to the 3' end of transcripts and do not span the entire gene. Thus, because antisense-strand transcripts commonly have a different splice structure they may not be detected. Subsequently the 110 candidate sense-antisense transcript pairs and the 80 potentially antisense-specific transcripts that were identified are likely to under-represent the number of true transcript pairs. In future studies, custom microarrays containing probes for sense and antisense transcripts would be useful as different target preparation assays would not be required, but because we aimed to obtain a broad representation of the extent of antisense transcription we chose to use the most comprehensive Wheat Genome Array.

The function of antisense-strand transcription is widely believed to regulate the expression of sense-strand transcripts at either transcription, mRNA maturation or translation [2]. In fact, Lapidot and Pilpel [1] reviewed the literature and postulated four mechanisms of action; transcriptional interference, RNA masking, double-stranded RNA (ds-RNA)-dependent mechanisms, and chromatin remodeling. The ds-RNA mechanisms would likely be the result of RNA-dependent RNA polymerases, which generate ds-RNA that are the precursors of short interfering RNA (siRNA). The timing of sense- and antisense-strand transcription is also important; for example, if the sense-strand is transcribed first up to a certain level followed by transcription of the antisense-strand, the biological result would be delayed inhibition of the sense-strand gene expression. Conversely, if the antisense-strand was transcribed first, this would result in pre-inhibition of sense-strand gene expression up to a threshold. Differences in the half-life of the sense-and antisense-strand transcripts, as well as tissue-specificity and potential light and/or diurnal transcript regulation [24] would also affect these scenarios. In the present study the timing of transcription and relative level of sense- and antisense-transcripts could not be determined because a single time-point was used for RNA extraction in each tissue, and the design of the assay did not allow valid comparisons between the 3'IVT and WT results to estimate transcript levels. Thus the mode of action of the detected sense-antisense transcript pairs would require further study.

An important observation in this study was the functional annotation of the sense-antisense transcript pairs, which indicated a significant over-representation of those involved in energy production, particularly photosynthesis. Additionally, many transcripts for ribosomal proteins involved in protein synthesis were identified. The abundance of antisense transcripts for these common plant processes may indicate that they are negatively regulated by antisense transcripts. Alternatively, the antisense transcripts could possibly be the result of ectopic expression. There is little data on large-scale antisense transcription profiling in plants to compare these results with, but a study in rice of leaf and seed tissue using Serial Analysis of Gene Expression (SAGE) identified sense-antisense transcript pairs and also found that the most abundant pairs were annotated as involved in energy production, including RUBISCO and a Photosystem I protein [11]. The similarity between studies shows that transcripts involved in photosynthesis are likely to be controlled by antisense transcripts in plants. An appealing explanation is the possibility for diurnal regulation of photosynthesis through antisense regulation. Although this study did not span a time-course required to demonstrate diurnal regulation, the results warrant further exploration of this hypothesis.

The results of the strand-specific RT-PCR also showed that antisense transcription is likely to be tissue-specific. Only one of the RT-PCR results was not in complete agreement with the microarray result, which could be due to truncated antisense transcripts where the priming sites were absent. In their microarray study of human cell antisense transcription, Ge *et al*. [18] found that 26% of the RT-PCR results were not consistent with microarray observations. In this study we also identified 80 transcripts as potentially antisense-specific, although further studies would be needed to confirm this because of the possibility for incorrectly oriented probes or strand bias during hybridization. The majority of these transcripts were annotated as unknown, but of those that were there was again a trend towards function in photosynthesis. A high percentage were also functionally involved in controlling transcription, including transcripts with homology to DNA-directed RNA polymerase, which indicates that gene expression in wheat may be regulated by antisense transcripts at the transcriptional level.

A recent study in wheat involving SAGE of developing grain also identified antisense transcripts [14], where the most abundant functional categories aside from unknown tags were associated with storage and reproduction. The abundance of these functional categories was due to the sampling of developing grain tissue, while the abundance of energy-related transcripts in our study is most likely due to the selection of photosynthetic tissues. For this reason, these two studies complement each other well. As in our study, Poole *et al*. [14] found that most antisense tags were of unknown function and that many transcripts were highly expressed in both sense and antisense, which may suggest a function of the antisense transcript for mediating alternative polyadenylation rather than down-regulation of the sense transcript, although there is no evidence for this at this stage. One other similarity to our study was the identification by Poole *et al*. [14] of antisense transcripts related to transcription, such as nucleotide binding proteins, which the authors suggest may enable the control of multiple pathways that require large scale changes during development. Other than these similarities, the results of our study differ from Poole *et al*. [14], which again is likely due to the complementary tissues analyzed.

This study was exploratory and revealed that the method was successful in identifying sense-antisense transcript pairs using the commercial Wheat Genome Array. The next step from this study is to select potentially interesting antisense transcripts for further study. There were several transcript pairs belonging to functional categories including 'Cell death' and 'Transcription' that may be involved in the regulation of important biological processes, and the antisense-specific transcripts related to transcription are also of interest. An understanding of the role of antisense transcription as it relates to gene expression may be important for the expression of certain phenotypes of interest. Additionally, knowledge of natural antisense transcripts may also be important for altering gene expression through transgenic studies in plants. The abundance of antisense-strand transcripts in plants is supported by recent studies using 'open' transcriptomics systems including SAGE [11,14] and Massively Parallel Signature Sequencing (MPSS) [12]. With the advent of RNA-Seq (RNA sequencing), which is high-throughput transcriptome sequencing method [25] that incorporates the use of next-generation sequence-by-synthesis technologies, the future will see a greatly enhanced discovery and understanding of antisense-strand transcription in plants.

## Conclusion

This study demonstrated the novel use of an adapted labeling protocol and a 3'IVT Affymetrix GeneChip microarray for large-scale identification of antisense transcription in wheat, a crop of great economic importance. The results show that antisense transcription is relatively abundant in wheat, and may affect the expression of valuable agronomic phenotypes. Strand-specific RT-PCR validated the microarray observations, and showed that antisense transcription is likely to be tissue specific. Most of the identified sense-antisense transcript pairs were annotated as genes involved in energy production, indicating that photosynthesis is likely to be under regulation by antisense transcripts.

## Methods

### Plant material and RNA extraction

The spring wheat genotype 'Chinese Spring' was selected for this study because the majority of GeneChip Wheat Genome Array (Affymetrix, Santa Clara, California, USA) probe sequences were based on its DNA sequence. Five tissue types were selected for this study; i.Germinated seed (germinated on wetted filter paper in a petri-dish in the dark for two days, radicle and plumule emerged), ii.Shoot (Feekes 1.2, emergence with second leaf unfolded), iii.Flag leaf (Feekes 9.0, flag leaf stage with ligule emerged), iv.Spike pre-anthesis (Feekes 10.5, heading complete), and v.Spike post-anthesis (Feekes 10.5.3, flowering complete to base of spike). For each tissue type except 'Germinated seed', six seeds were planted in 6-inch round pots using a potting mix (6 peat moss: 4 vermiculite with lime: 3 sand: 3 commercial potting mix: 2 perlite: 1.7 g/L lime: 3.3 g/L Osmocote: 2.2 g/L ammonium nitrate). Seedlings were grown to the appropriate growth stages for tissue collection in a greenhouse with a stable temperature of 25 ± 2°C and a 16 h light/8 h dark cycle. All tissue samples were immediately frozen in liquid nitrogen, and total RNA was extracted from 1.0 g of each pooled tissue type using the Trizol^® ^Plus RNA Purification Kit (Invitrogen, Carlsbad, CA) with an on-column DNase treatment. Purified total RNA samples were quantified with a NanoDrop^® ^ND-1000 (NanoDrop, Wilmington, DE) spectrophotometer, and satisfactory purity was indicated by A260:280 ratios of 1.9–2.1 in 10 mM Tris-Hcl (pH 7.5). Integrity of total RNA samples was assessed by denaturing formaldehyde gel electrophoresis, where the presence of sharp 28S and 18S ribosomal RNA bands at an intensity ratio of ~2:1 (28S:18S) indicated good integrity. The five total RNA samples were then mixed at equal concentrations before analysis.

### Wheat Genome Array

The GeneChip Wheat Genome Array (Affymetrix, Santa Clara, California, USA) is a 3'IVT array that includes 61,127 probe sets representing 55,052 transcripts for all 21 wheat chromosomes in the genome. 59,356 probes sets represent modern hexaploid (A, B and D genomes) bread wheat (*T. aestivum*) and are derived from the public content of the *T. aestivum *UniGene Build #38 (April 24, 2004). 1,215 probe sets are derived from ESTs of a diploid near relative of the A genome (*T. monococcum*), a further 539 represent ESTs of the tetraploid (A and B genomes) macaroni wheat species *T. turgidum*, and five are from ESTs of a diploid near relative of the D genome known as *Aegilops tauschii*. Probe sets consisted of pairs of 11 perfect match (PM) and mismatch (MM) 25-mer oligonucleotides designed from the 3' end of exemplar sequences, with nucleotide 13 as the MM. Array annotation information is available on the NetAffx data analysis center .

### Sense-strand transcription analysis

The 3'IVT Wheat Genome Array detects sense strand transcription by generating antisense-orientated labeled complementary RNA (cRNA) from the original RNA sample that is then hybridized to the probes that are designed to hybridize to the antisense-orientated labeled cRNA. Although the system generates antisense-orientated labeled cRNA, the assayed strand for transcription is the sense strand. Thus, to assay sense strand transcription in the mixed Chinese Spring total RNA sample, the regular 3'IVT Affymetrix protocol was carried out . Briefly, double-stranded cDNA was generated from mRNA using a T7-oligo(dT) primer. The double-stranded cDNA was cleaned up and used as template for *in vitro *transcription (IVT) in the presence of T7 RNA Polymerase and a biotinylated nucleotide analog/ribonucleotide mix for cRNA amplification and biotin labeling. The biotinylated cRNA target was then cleaned up, fragmented, and hybridized to the Wheat Genome Array. All hybridizations and data acquisition was performed at the Genomics Core Facility at Washington State University (Pullman, Washington, USA) according to standard Affymetrix protocols .

### Antisense-strand transcription analysis

To assay antisense strand transcription, the Affymetrix Whole Transcript (WT) Sense Target Labeling Assay  was used. This WT assay is intended for use on Affymetrix Whole Transcript expression arrays, which contain probes designed to hybridize with sense-orientated labeled cDNA. Because the probes of the 3'IVT Wheat Genome Array are designed to hybridize to antisense-orientated labeled cRNA derived from sense strand transcription, the sense-orientated labeled cDNA generated by the WT assay will not hybridize to the array unless it was derived from antisense transcription. Thus to discover antisense transcription in the mixed Chinese Spring total RNA sample, the target was prepared using the WT assay but hybridized to the 3'IVT Wheat Genome Array. Briefly, double-stranded cDNA was synthesized with random hexamers coupled to the T7 promoter, followed by IVT amplification with T7 RNA polymerase to produce cRNA. A second cycle cDNA synthesis was then performed using random primers for reverse transcription, which converted the cRNA into single-stranded cDNA in the same orientation as the original mRNA (sense-orientation). The single-stranded cDNA was then cleaned up, fragmented, and hybridized to the Wheat Genome Array according to standard Affymetrix protocol.

### Data analysis

Using GeneChip Operating Software (GCOS) v.1.4 (Affymetrix, Santa Clara, California, USA), image quality control was performed by inspecting raw intensity (DAT) files for scratches/smears and uniform performance of the B2 oligo around the border of each image. Data quality control was from raw data in CEL files using the *affyQCreport *package [22] of Bioconductor [23], which provided Affymetrix recommended quality metrics, per array intensity distributions, between array comparisons, and other diagnostic plots for each hybridization. The Bioconductor [23] package *affy *[26] was used to read in the raw Affymetrix 'CEL' files, which were pre-processed using Robust Multi-array Average (RMA) [27,28]. Pre-processing was modified so that only expression value summarization was applied. Background correction and normalization were omitted because the arrays were hybridized using different labeling assays. PMA (present, marginal and absent) calls were calculated for each probe set using a Wilcoxon rank sum test from MAS 5.0 [29]. Only those probe sets with a Wilcoxon *p*-value < 0.01 were considered 'present'.

Probe sets called as present were also required to possess a summarized log_2 _expression value greater than the *bioB *spiked-in hybridization control (>8.76 in the 3'IVT assay and >9.86 in the WT assay). Probe sets meeting these criteria were annotated using HarvEST (Affymetrix Wheat1 Chip version 1.52), which identified the corresponding unigene for each probe set and provided the current best BLASTX hit from the non-redundant (nr) database of NCBI, as well as the best BLASTX hits from rice and *Arabidopsis thaliana *TIGR databases . A database hit <1e-10 was considered as significant, otherwise the unigene was annotated as 'no homology'. Unigenes were assigned to functional categories based on Munich Information Center for Protein Sequences (MIPS; ) classifications. All minimum information about microarray experiments (MIAME) guidelines were observed and GeneChip data was deposited into WheatPLEX [30] accession TA21, as well as NCBI's Gene Expression Omnibus [31] accession number GSE12528. For gene ontology (GO), the rice locus matching each probe set in the HarvEST output provided the most comprehensive annotation set. To assess the significance of energy-related transcripts, common terms in the rice transcript description for energy-related transcripts were selected and used as search terms across the annotation of transcripts found to be present in one or both assays. The search terms used were: photosystem, ribosomal protein, chloroplast, chlorophyll, cp12, oxygen-evolving, carbonic anhydrase, ATP synthase, ribulose, cytochrome, NADH. Each probe set was inspected as to whether or not it contained one or more the 'energy' search terms. To assess whether 'energy' related transcripts were significantly overrepresented in the identified sense-antisense transcript pairs than was expected by random chance, we performed a hypergeometric test.

### Strand-specific transcription validation

Ten probe sets that were found to be transcribed on both strands were selected for validation using strand-specific reverse transcription-PCR (RT-PCR) (Table 2). The strand-specificity of the Qiagen One Step RT-PCR kit (Qiagen, Valencia, California, USA) has been confirmed in previous studies [32], thus was selected for use in this study. DNase-treated total RNA from each tissue type was used to determine potential tissue specificity of transcription. Unigene sequences for each of the 10 probe sets were identified using HarvEST (Affymetrix Wheat1 Chip version 1.52), and primer pairs were designed using Vector NTI (v. 10.3.0, Invitrogen Corporation). Strand-specificity was achieved by selective use of primers in the reverse transcription step, where the reverse primer was used to detect sense transcripts, and the forward primer to detect antisense transcripts. PCR reactions following reverse transcription were carried out in the presence of both forward and reverse primers, with the following cycling parameters; i. 50°C for 60 min (reverse transcription), ii. 95°C for 15 min (activate polymerase and deactivate RT enzymes), iii. 4°C for 5 min (added missing primer/s for PCR at this point), iv. 94°C for 30 s, 60°C for 30 s, 72°C for 45 s (PCR cycling repeated 35 times), and v. 72°C for 10 min (final extension). All PCR products were visualized on 1.5% agarose gels.

## Authors' contributions

TEC carried out the tissue collection, RNA extraction, RT-PCR, data analysis, and drafted the manuscript. MLS participated in the design of the study and performed the statistical analysis. TEC and XC conceived the study. XC participated in its design and coordination and helped to draft the manuscript. All authors read and approved the final manuscript.

## Supplementary Material

Additional file 1 **List of the 110 sense-antisense transcript pairs with Present calls (*P *< 0.01), >8.76 3'IVT assay log_2 _expression value and >9.86 WT assay log_2 _expression value**. The data provided represents annotation and statistical values for the candidate sense-antisense transcript pairs. Click here for file

Additional file 2 **List of the 80 potential antisense-specific transcripts with Present calls (*P *< 0.01) and >9.86 WT assay log_2 _expression value**. The data provided represents annotation and statistical values for the potential antisense-specific transcripts. Click here for file
